# Antimicrobial nodule-specific cysteine-rich peptides disturb the integrity of bacterial outer and inner membranes and cause loss of membrane potential

**DOI:** 10.1186/s12941-016-0159-8

**Published:** 2016-07-28

**Authors:** Kata R. Mikuláss, Krisztina Nagy, Balázs Bogos, Zsolt Szegletes, Etelka Kovács, Attila Farkas, György Váró, Éva Kondorosi, Attila Kereszt

**Affiliations:** 1 Institute of Biochemistry, Hungarian Academy of Sciences, Temesvári körút 62, 6726 Szeged, Hungary; 2 Institute of Biophysics of the Biological Research Centre, Hungarian Academy of Sciences, Temesvári körút 62, 6726 Szeged, Hungary; 3 Department of Environmental Systems Science, Institute of Integrative Biology, Group of Theoretical Biology, ETH Zürich, Universität strasse 16, CHN K18, 8092 Zurich, Switzerland

**Keywords:** Antimicrobial activity, Membrane disruption, Atomic force and scanning electron microscopy

## Abstract

**Background:**

Certain legume plants produce a plethora of AMP-like peptides in their symbiotic cells. The cationic subgroup of the nodule-specific cysteine-rich (NCR) peptides has potent antimicrobial activity against gram-negative and gram-positive bacteria as well as unicellular and filamentous fungi.

**Findings:**

It was shown by scanning and atomic force microscopies that the cationic peptides NCR335, NCR247 and Polymyxin B (PMB) affect differentially on the surfaces of *Sinorhizobium meliloti* bacteria. Similarly to PMB, both NCR peptides caused damages of the outer and inner membranes but at different extent and resulted in the loss of membrane potential that could be the primary reason of their antimicrobial activity.

**Conclusions:**

The primary reason for bacterial cell death upon treatment with cationic NCR peptides is the loss of membrane potential.

**Electronic supplementary material:**

The online version of this article (doi:10.1186/s12941-016-0159-8) contains supplementary material, which is available to authorized users.

## Findings

One of the greatest challenges to fight bacterial infections in the medical practice is to find an antibiotic that can eliminate multidrug resistant pathogens [1]. Therefore, identification of novel antimicrobial agents that have different bacterial targets from those of classical antibiotics is necessary.

Among the potential antibiotic candidates are the antimicrobial peptides (AMPs) that are small, mostly cationic and ribosomally synthesized molecules produced by all living organisms [2]. AMPs can have antibacterial and antifungal activities. Some of them kill only a few species while others are active against both gram-negative and gram-positive bacteria as well as fungi [2]. Many AMPs with net cationic charge and amphipathic nature interact with the negatively charged bacterial membranes [3–5] leading to cell lysis caused by membrane disruption. Alternatively, AMPs may enter cells and interact with their intracellular targets interfering with DNA, RNA, protein or cell wall synthesis [6–9].

Extremely rich sources of AMPs are the plants where up to several hundreds of peptide-coding genes can be expressed in specific organs constitutively or induced locally or systematically by the attack of pathogenic microbes. Interestingly, the AMP-like nodule-specific cysteine-rich (NCR) peptides play important role in the mutualistic nitrogen-fixing symbiosis of certain leguminous plants with rhizobia resulting in the formation of root nodules where plant cells contain thousands of intracellular endosymbionts. In *Medicago truncatula* nodule cells infected with *Sinorhizobium meliloti*, hundreds of NCR peptides are produced which direct irreversible differentiation of the bacteria into large polyploid nitrogen-fixing bacteroids [10–13]. Over 600 potential NCR peptides are predicted from the *M. truncatula* genome sequence [14] and almost 150 different NCR peptides have been detected in isolated bacteroids by mass spectrometry [15]. NCRs are characterized by a relatively conserved secretory signal peptide (SP) and highly variable amino acid sequence and isoelectric point of the mature peptide where positions of four or six cysteines are conserved. The structure of NCRs resembles that of defensins, the most abundant plant innate immunity effectors, that have also a SP and a variable, usually cationic mature peptide, however with eight cysteines [7]. Similarly to defensins, synthetic cationic NCR peptides with pI > 9 have antimicrobial activities while neutral and anionic ones, such as NCR001, are inactive. For example, NCR247 (pI = 10.15) and NCR335 (pI = 11.22) are both effective against gram-negative and gram-positive bacteria [16] as well as fungi [17], however their spectrum of activity is not identical (see [1] and Additional file 1) suggesting that in addition to the net positive charge, the amino acid composition and sequence contribute also to their activities. Investigation of NCR247 and NCR335 treated *Escherichia coli* cells by atomic force microscopy (AFM) revealed increased surface roughness suggesting the damage of the cell envelope [18].

**Fig. 1:**
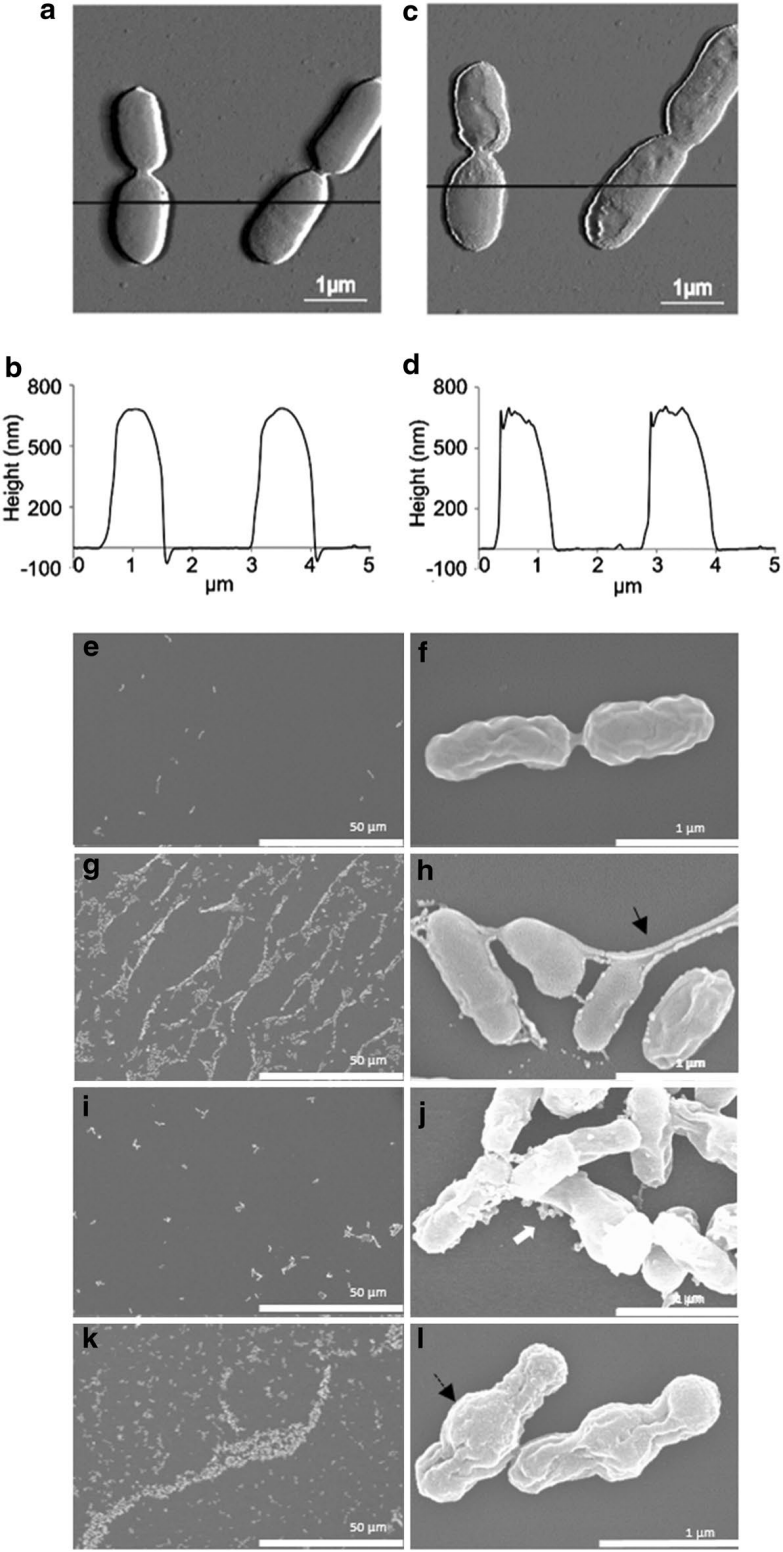
The effect of peptides on the morphology of *S. meliloti*. Images (**a**, **b**) and height measurement data (**c**, **d**) obtained by atomic force microscopy before (**a**, **c**) and after (**b**, **d**) NCR247 treatment reveal surface roughness caused by the peptide. Scanning electron micrographs of untreated cultures (**e**, **f**) as well as cultures treated with NCR247 (**g**, **h**), NCR335 (**i**, **j**) or PMB (**k**, **l**) at 25 µg/ml for 30 min show cell aggregation (**g**, **k**) where cells are connected with thread-like structures (**h**) or have swollen middle part (**l**) indicated by *arrows*

In this study, we investigated how NCR247 and NCR335 affect the cell surface as well as the outer and inner membranes (OM and IM respectively) of the α-Proteobacterium *S. meliloti*, the natural target of the peptides. We compared the effect of these NCR peptides to that of Polymyxin B (PMB), which alters bacterial outer membrane permeability and then disrupts the cytoplasmic membrane of gram-negative bacteria [19]. Moreover, we used also the negatively charged peptide, NCR001 as a control possessing no antimicrobial activity. NCR247 and NCR335 inhibited the growth of rhizobia at 25 and 12 µg/ml (Minimal Inhibitory Concentration), respectively, in broth microdilution assays, and were able to decrease the number of living cells by two and four orders of magnitude, respectively, when they were used at 50 µg/ml concentration in phosphate buffer for three hours. In other—including clinically relevant—bacteria, the minimal bactericid concentration of the peptides varied between 20 and 125 µg/ml (Additional file 1). High-resolution AFM images of immobilized *S. meliloti* cells after treatment with 25 µg/ml of NCR247 revealed no change in the average height (~600 nm) of bacteria, while a clear difference was observed in the roughness of the cell surface (Fig. 1a–d). *S. meliloti* has a smooth curved surface (Fig. 1a, b), however, addition of the NCR247 peptide for 1 h increased the surface roughness (Fig. 1c, d) while no surface alterations occurred in the mock- and NCR001-treated cells. Prolonging the treatment for 3 h caused no further changes, cells treated for 1 and 3 h were alike. These observations were in line with reported surface corrugation of the *E. coli* cell envelope by NCR247 [18]. Similar study on the NCR335 treated *S. meliloti* cultures could not be performed as the bacteria lost their attachment to the poly-l-lysine coated muscovite mica surface.

The differences in the effect of the two peptides on the bacterial cell envelop were further observed by scanning electron microscopy (SEM) (Fig. 1e–l). The treatment with the NCR247 peptide caused cell aggregation and the formation of large, network-like creations (Fig. 1g, h), while cells treated with NCR335 remained separated or formed small aggregates (Fig. 1i, j). Higher magnifications revealed that the NCR247-treated cells are connected with thread-like structures that seem to be formed by materials released from destabilized cell surfaces (Fig. 1h). In contrast, the majority of the NCR335 treated cells were collapsed and emptied, while from the other cells vesicular structures were released that might indicate the outflow of the cell content before collapsing of the cells (Fig. 1j). PMB provoked also the aggregation of *S. meliloti* cells but networks were not formed and the middle part of bacteria showed swelling (Fig. 1k–l).

**Fig. 2:**
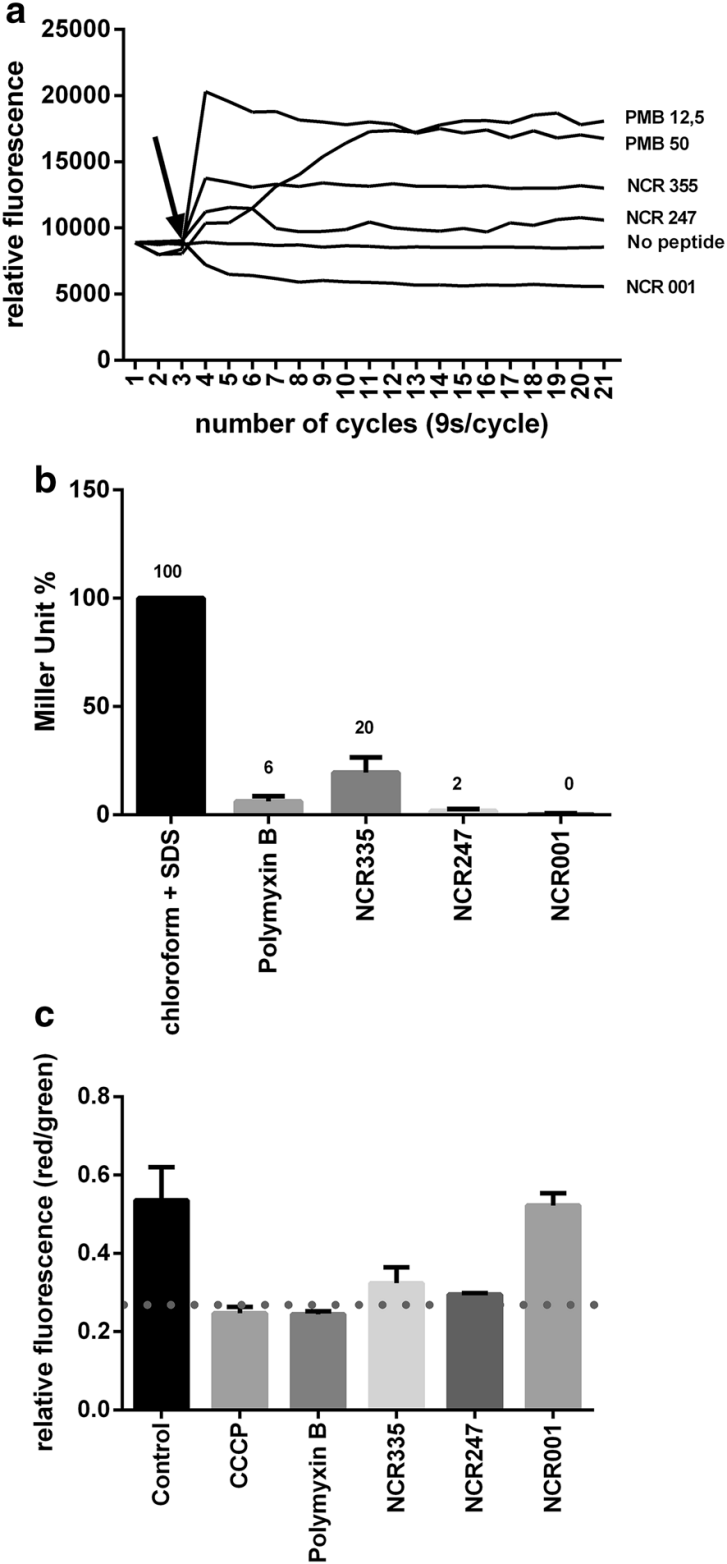
The effect of NCR peptides and PMB on membrane integrity of *S. meliloti.* **a** Outer membrane permeability measured by the fluorescence of NPN at 50 µg/ml (NCR335: 6.4 µM; NCR247: 16.6 µM; NCR001: 9.5 µM) peptide concentrations, and at 12.5 (9 µM) and 50 µg/ml (36 µM) for PMB. **b** Inner membrane permeability measured by β-galactosidase activity at 50 µg/ml peptide concentrations. **c** Membrane potential of the *S. meliloti* cells measured by the *red*/*green* fluorescence ration of DiOC_2_(3). This dye “exhibits green fluorescence in low concentration in all bacterial cells, however, it accumulates and self-associates in cells that are maintaining a membrane potential resulting in the fluorescence emission to shift from *green* to *red*” (Thermo Fisher Scientific)

The changes in the surface and the shape of the bacteria observed by the microscopic studies may have been triggered by the effects of the peptides on the bacterial membranes, however, it remained unclear whether and how NCR247 and NCR335 affect the integrity and permeability of OM and IM. The integrity of the OM can be tested with the hydrophobic 1-*N*-phenylnaphthylamine (NPN) probe which cannot enter the intact OM but can pass the destabilized one and by entering the phospholipid layer gives rise to strong fluorescence [20]. PMB as expected but also NCR335 and to lesser extent NCR247 treatment of *S. meliloti* resulted in NPN fluorescence (Fig. 2a) indicating the damage of the OM by these peptides. Yet, the extent and the kinetics of OM damage were different and dependent on the peptide concentrations (Additional file 2). PMB provoked the most pronounced effect, however with a slower kinetics at lower concentrations. NCR335 was equally efficient at 50, 25 and 12.5 µg/ml but provoked a weaker OM permeabilization than PMB with the same or comparable kinetics. NCR247 caused only a mild damage at 50 µg/ml and even weaker at 25 µg/ml and had no effect at lower concentrations. NCR001 (pI = 5.01) did not increase the OM permeability suggesting that binding of cationic NCRs to a negatively charged site in the lipopolysaccharide layer could be responsible for the OM permeability.

To investigate the possible damage to the IM we took advantage of the fact that the IM is not permeable for ortho-nitrophenyl-β-galactoside (ONPG), the artificial substrate of the cytoplasmic β-galactosidase enzyme, thus, the enzyme activity can be measured only after the disruption of the membrane [21]. Treatment of *S. meliloti* cells expressing constitutively the *lacZ* gene with the cationic peptides resulted in measurable β-galactosidase activity (Fig. 2b) that was 2–20 % of the total enzyme activity obtained after disrupting the cells with SDS and chloroform. In contrast to the OM, the IM disruption was more effective by NCR335 than PMB even when PMB was used in ~sixfold higher molar concentration. The IM damage was further confirmed by measuring the membrane potential of the peptide treated cells using the fluorescent membrane-potential indicator dye, DiOC_2_(3), provided in the BacLight™ Bacterial Membrane Potential Kit (Thermo Fisher Scientific) (Fig. 2c) [22]. Likewise carbonyl cyanide m-chlorophenyl hydrazone (CCCP), a known protonophore, PMB, NCR247 and NCR335 caused the loss of membrane potential that was not observed when the cells were treated by NCR001.

To conclude, we have shown that the cationic symbiotic peptides, NCR247 and NCR335 destabilize and disrupt the integrity of the cell envelope acting both on the outer and inner membranes of the bacteria, as they disrupt the cytoplasmic membrane of fungi, too [17]. Their effects are similar but not identical to each other and to PMB. Their antibacterial properties are due to the loss of membrane potential leading to the inhibition of cellular processes and cell death, however, we cannot exclude that the peptides have intracellular targets, as it was shown for NCR247 [23]. Their broad-spectrum antimicrobial property [16, 17] and low cytotoxicity [17] qualify cationic NCRs as potential therapeutic compounds, however, their use may be limited as systemic agents because of the inhibition of their activity by bivalent cations and serum [12, 16, 17].

## Additional files

10.1186/s12941-016-0159-8 Minimal Bactericid concentration of the NCR247 and NCR335 peptides on different gram-positive and gram-negative bacteria.
10.1186/s12941-016-0159-8 The concentration dependence of the hydrophobic 1-*N*-phenylnaphthylamine (NPN) probe. The outer membrane permeability measured (A) at 50 µg/ml; (B) at 25 µg/ml; (C) at 12.5 µg/ml; and (D) at 6.25 µg/ml peptide concentrations.
10.1186/s12941-016-0159-8 Description of the methods used.

## Abbreviations

NCR: nodule-specific cysteine-rich peptides
AMPs: antimicrobial peptides
SP: signal peptide
PMB: polymyxin B
MIC: minimal inhibitory concentration
MBC: minimal bactericid concentration
AFM: atomic force microscopy
SEM: scanning electron microscopy
OM: outer membrane
IM: inner membrane
NPN: 1-*N*-phenylnaphthylamine
ONPG: ortho-nitrophenyl-β-galactoside
DiOC_2_(3): 3,3′-diethyloxa-carbocyanine iodide
CCCP: carbonyl cyanide m-chlorophenyl hydrazone

## References

[1] Yoshikawa TT (2002). Antimicrobial resistance and aging: beginning of the end of the antibiotic era?. J Am Geriatr Soc.

[2] Maroti G, Kereszt A, Kondorosi E, Mergaert P (2011). Natural roles of antimicrobial peptides in microbes, plants and animals. Res Microbiol.

[3] Vaara M, Vaara T (1983). Polycations as outer membrane-disorganizing agents. Antimicrob Agents Chemother.

[4] Teixeira V, Feio MJ, Bastos M (2012). Role of lipids in the interaction of antimicrobial peptides with membranes. Prog Lipid Res.

[5] Hancock RE, Chapple DS (1999). Peptide antibiotics. Antimicrob Agents Chemother.

[6] Brogden KA (2005). Antimicrobial peptides: pore formers or metabolic inhibitors in bacteria?. Nat Rev Microbiol.

[7] Ganz T (2003). Defensins: antimicrobial peptides of innate immunity. Nat Rev Immunol.

[8] Hale JD, Hancock RE (2007). Alternative mechanisms of action of cationic antimicrobial peptides on bacteria. Expert Rev Anti Infect Ther.

[9] Hancock RE, Sahl HG (2006). Antimicrobial and host-defense peptides as new anti-infective therapeutic strategies. Nat Biotechnol.

[10] Mergaert P, Nikovics K, Kelemen Z, Maunoury N, Vaubert D, Kondorosi A, Kondorosi E (2003). A novel family in Medicago truncatula consisting of more than 300 nodule-specific genes coding for small, secreted polypeptides with conserved cysteine motifs. Plant Physiol.

[11] Mergaert P, Uchiumi T, Alunni B, Evanno G, Cheron A, Catrice O, Mausset AE, Barloy-Hubler F, Galibert F, Kondorosi A (2006). Eukaryotic control on bacterial cell cycle and differentiation in the Rhizobium-legume symbiosis. Proc Natl Acad Sci USA.

[12] Van de Velde W, Zehirov G, Szatmari A, Debreczeny M, Ishihara H, Kevei Z, Farkas A, Mikulass K, Nagy A, Tiricz H (2010). Plant peptides govern terminal differentiation of bacteria in symbiosis. Science.

[13] Horvath B, Domonkos A, Kereszt A, Szucs A, Abraham E, Ayaydin F, Boka K, Chen Y, Chen R, Murray JD (2015). Loss of the nodule-specific cysteine rich peptide, NCR169, abolishes symbiotic nitrogen fixation in the Medicago truncatula dnf7 mutant. Proc Natl Acad Sci USA.

[14] Young ND, Debelle F, Oldroyd GE, Geurts R, Cannon SB, Udvardi MK, Benedito VA, Mayer KF, Gouzy J, Schoof H (2011). The Medicago genome provides insight into the evolution of rhizobial symbioses. Nature.

[15] Durgo H, Klement E, Hunyadi-Gulyas E, Szucs A, Kereszt A, Medzihradszky KF, Kondorosi E (2015). Identification of nodule-specific cysteine-rich plant peptides in endosymbiotic bacteria. Proteomics.

[16] Tiricz H, Szucs A, Farkas A, Pap B, Lima RM, Maroti G, Kondorosi E, Kereszt A (2013). Antimicrobial nodule-specific cysteine-rich peptides induce membrane depolarization-associated changes in the transcriptome of *Sinorhizobium meliloti*. Appl Environ Microbiol.

[17] Ordogh L, Voros A, Nagy I, Kondorosi E, Kereszt A (2014). Symbiotic plant peptides eliminate Candida albicans both in vitro and in an epithelial infection model and inhibit the proliferation of immortalized human cells. Biomed Res Int.

[18] Nagy K, Mikulass KR, Vegh AG, Kereszt A, Kondorosi E, Varo G, Szegletes Z (2015). Interaction of cysteine-rich cationic antimicrobial peptides with intact bacteria and model membranes. Gen Physiol Biophys.

[19] Rosenthal KS, Storm DR (1977). Disruption of the *Escherichia coli* outer membrane permeability barrier by immobilized polymyxin B. J Antibiot (Tokyo).

[20] Trauble H, Overath P (1973). The structure of *Escherichia coli* membranes studied by fluorescence measurements of lipid phase transitions. Biochim Biophys Acta.

[21] Oliva B, Gordon G, McNicholas P, Ellestad G, Chopra I (1992). Evidence that tetracycline analogs whose primary target is not the bacterial ribosome cause lysis of *Escherichia coli*. Antimicrob Agents Chemother.

[22] Plasek J, Sigler K (1996). Slow fluorescent indicators of membrane potential: a survey of different approaches to probe response analysis. J Photochem Photobiol B.

[23] Farkas A, Maroti G, Durgo H, Gyorgypal Z, Lima RM, Medzihradszky KF, Kereszt A, Mergaert P, Kondorosi E (2014). Medicago truncatula symbiotic peptide NCR247 contributes to bacteroid differentiation through multiple mechanisms. P Natl Acad Sci USA.

