# Supplementary material for: Antimicrobial nodule-specific cysteine-rich peptides disturb the integrity of bacterial outer and inner membranes and cause loss of membrane potential
Source: Ann Clin Microbiol Antimicrob. 2016 Jul 28;15:43. doi: 10.1186/s12941-016-0159-8 (PMC4964015; doi:10.1186/s12941-016-0159-8)
Supplement: Supplementary file 1 — 10.1186/s12941-016-0159-8 Minimal Bactericid concentration of the NCR247 and NCR335 peptides on different gram-positive and gram-negative bacteria. [file 12941_2016_159_MOESM1_ESM.pdf]

	MBC	
	**NCR247**	**NCR335**
Sinorhizobium meliloti AK631	100 µg/ml	50 µg/ml
Escherichia coli K12	125 µg/ml	50 µg/ml
Salmonella typhimurium	100 µg/ml	50 µg/ml
Pseudomonas aeruginosa	100 µg/ml	70 µg/ml
Lactobacillus plantarum	40 µg/ml	30 µg/ml
Listeria monocytogenes	30 µg/ml	20 µg/ml
Staphylococcus aureus	125 µg/ml	30 µg/ml

Additional File 1: Minimal Bactericid concentration of the NCR247 and NCR335 peptides on different Gram-positive and Gram-negative bacteria
