# Supplementary material for: Antimicrobial nodule-specific cysteine-rich peptides disturb the integrity of bacterial outer and inner membranes and cause loss of membrane potential
Source: Ann Clin Microbiol Antimicrob. 2016 Jul 28;15:43. doi: 10.1186/s12941-016-0159-8 (PMC4964015; doi:10.1186/s12941-016-0159-8)
Supplement: Supplementary file 3 — 10.1186/s12941-016-0159-8 Description of the methods used. [file 12941_2016_159_MOESM3_ESM.docx]

**METHODS**

***Antimicrobial peptides***

NCR247 (NH2-RNGCIVDPRCPYQQCRRPLYCRRR; MW = 3.0 kDa; pI = 10.15) and NCR335 (NH2–RLNTTFRPLNFKMLRFWGQNRNIMKHRGQKVHFSLILSDCKTNKDCPKLRRANVRCRKSYCVPI;

MW = 7.7 kDa; pI = 11.22) peptides were chemically synthetized and provided by ProteoGenyx Inc. (France) in a high (>95%) purity.

***Atomic force microscopy***

Details of the measurements and sample preparations are described in Reference 18.

Nagy K, Mikulass KR, Vegh AG, Kereszt A, Kondorosi E, Varo G, Szegletes Z: **Interaction of cysteine-rich cationic antimicrobial peptides with intact bacteria and model membranes**. *Gen Physiol Biophys* 2015, **34**(2):135-144

***Scanning electron microscopy***

Exponentially growing cultures of bacteria were collected, washed twice and suspended 10 mM potassium phosphate buffer (pH=7) and treated with peptides at 30 °C for 30 minutes. After peptide treatment, the cells were fixed with 2.5% glutaraldehyde dissolved in cacodylate buffer, washed with 10 mM potassium phosphate buffer (pH=7) and were dehydrated in ethanol of increasing concentration (50%-70%-80%-90%-95%-98%-100%). As the last step of the dehydration, the 100% ethanol was replaced by tert-butanol in which the cells were kept at 4 °C overnight. The samples were freeze-dried, covered by gold and investigated with a HITACHI S-4700 scanning electron microscope.

***Measuring the NPN fluorescence***

The cells from exponentially growing cultures were collected, washed and suspended in 5 mM HEPES buffer (pH=7.2). 80 µl NPN of 25 µM concentration and 100 µl of bacterial suspension (OD_600_=0.1) were pipetted into the wells of a black 96-well microtiter plate and the fluorescence were measured for 3 cycles (9 seconds/cycle) in a FLUOstar OPTIMA plate-reader (BMG Labtech). After the three cycles, 20 µl of peptide was added and the fluorescence were measured for an additional 18 cycles.

***Measuring the β-galactosidase activity***

For the measurements, *Sinorhizobium meliloti* cells carrying the pXLDG4 plasmid (Leong, S. A., P. H. Williams és G. S. Ditta (1985). Analysis of the 5' Regulatory Region of the Gene for Delta-Aminolevulinic-Acid Synthetase of Rhizobium-Meliloti. Nucleic Acids Research 13: 5965-5976) providing constitutive β-galactosidase activity were used. The measurements were performed as described by Miller (Experiments in molecular genetics. (1972)).

***Measuring the membrane potential***

For the measurements, the BacLight™ Bacterial Membrane Potential Kit (Thermo Fisher Scientific) was used according to the instructions of the manufacturer.
